# Marking the inclusion of the *Korean Journal of Women Health Nursing* in PubMed Central and strategies to be promoted to a top-tier journal in the nursing category

**DOI:** 10.4069/kjwhn.2022.08.19

**Published:** 2022-09-30

**Authors:** Sun Huh

**Affiliations:** Department of Parasitology and Institute of Medical Education, College of Medicine, Hallym University, Chuncheon, Korea

## Celebrating the *Korean Journal of Women Health Nursing* for inclusion in PubMed Central

I heard from Dr. Sue Kim, the editor-in-chief of the *Korean Journal of Women Health Nursing* (KJWHN), that the PubMed Central (PMC) Full-Participation Agreement and PMC banner for the journal were dispatched to PMC, and PMC received them on July 30, 2022. I was thrilled to hear the news because this is the first case of PMC accepting a non-English life science journal published in Korea. Seeing a Korean-language journal be accepted by PMC has always been my goal since August 2006, when I succeeded in producing PMC XML files [1]. It is also an event worth celebrating because it is the second of more than 20 nursing journals in Korea to be included in PMC. The first journal was *Child Health Nursing Research*, which only publishes in English [2].

## Language policy of the journal

In retrospect, I was invited as a speaker at the 15th Summer Seminar of the Korean Society of Women Health Nursing on May 27, 2010. I suggested changing the journal’s language to English-only to add the journal to PMC. I made this suggestion because biomedical journals published in Korea began to be listed in PMC in 2008, and at the time, PMC only accepted English-language journals [1]. Any journals indexed in PMC are also searchable from PubMed, the largest free literature database that provides English abstracts of life science journals. This is the shortest way for a local nursing journal to become an international journal. The frequency of citations by articles published in other journals increases dramatically after being indexed in PMC, by two to 10 times [3]. Furthermore, inclusion in PMC leads to more frequent submissions from international researchers. However, this suggestion was not attempted because of the difficulty of recruiting English manuscripts. Most society members who wrote manuscripts in English submitted them to international journals. Therefore, English-only journal was not feasible for the Society.

However, PMC changed its language policy in 2019, and it now accepts non-MEDLINE, non-English journals. PMC began to review these journals if their primary content was largely in English. Although the exact amount required for “largely in English” is not fixed, more than half of the journal content would be a reasonable target.

KJWHN stated in June 2021 that it would continue to value Korean-language submissions to serve the Korean scholarly community while simultaneously offering high-quality English-language articles from researchers in Korea and abroad [4]. This statement was also accompanied by a marked increase in English-language articles, which surpassed the number of Korean-language articles. Aside from KJWHN, it is not easy to find non-MEDLINE, non-English language journals—including those published in Chinese, Japanese, French, and Spanish—that had been newly listed in PMC since 2020. The change of language policy in 2019 by PMC has greatly benefited non-English journal editors who are eager to promote theirs to international journals. I am hopeful that KJWHN’s success will encourage other non-English nursing journals.

## What is the meaning of a journal becoming a PMC journal?

KJWHN is the first case of a non-MEDLINE, non-English journal in Korea to be indexed in PMC. Its inclusion makes it possible to disseminate valuable nursing information on women’s health from Korea to the world. Another non-MEDLINE, non-English journal is at the forthcoming stage: *Taehan Yongsang Uihakhoe Chi* (https://www.ncbi.nlm.nih.gov/nlmcatalog/101479271). Therefore, these journals can serve as excellent examples for other non-MEDLINE, non-English journals in Korea. More than 20 Korean-language nursing journals are published in Korea, only two of which are published in English: *Asian Nursing Research and Child Health Nursing Research*. Although both journals are open-access, *Child Health Nursing Research* was only included in PMC in January 2022 [2]. Seeing these examples, those non-English nursing journal editors can have the confidence to add their journals to PMC.

## How long can a Korean-language journal survive?

The *Journal of the Korean Medical Association* (https://jkma.org/), published in Korean by the Korean Medical Association (KMA), may be able to survive for more than 100 years. The reason for this is that it is the official journal of the KMA, the official organization of all physicians in Korea, and the editorial board invites most articles. This journal’s scope mainly relates to education and training for self-employed physicians, general practitioners, medical residents, and the general public. However, the reality of many Korean-language journals is that the number of submissions has decreased, even for training purposes. In other words, researchers want to be recognized for a higher-valued achievement if they spend time writing papers. Therefore, it is challenging to recruit authors unless the journal is listed in international indexing databases. Korean-language life science journals have gradually converted to English-only since 2008 and are usually added to PMC. For the promotion of the journal to a top-tier international level, many editors have changed the journal’s language to English. However, KJWHN’s success in PMC inclusion has now shown that it is possible for Korean-language journals to be added to PMC if the amount of English articles is more than 50% per issue. The survival of Korean-language journals is essential to maintain Korean as a scientific language, although English has already been positioned as the main scientific language. Even while keeping the original language, expanding the number of English articles, although difficult, will be essential preparation for the promotion of academic society journals.

## International Asian nursing journals’ performance

There are 33 nursing journals published in Asia indexed in the following databases: PMC, MEDLINE, Scopus, Web of Science Core Collection (SCIE and ESCI) (Supplementary material 1). Out of them, 12 journals are published in Korea; seven journals in Iran; three journals in Indonesia; two each from China, Taiwan, and Turkey; and one each from Japan, Hong Kong, Philippines, India, and Thailand. Out of these 33 journals, the 12 PMC or MEDLINE journals will show outstanding performance because they are searchable in PubMed. In PMC or PubMed, articles’ quality is the most critical factor determining whether they will be cited by other researchers. Journal brand is not a consideration by researchers in citing articles. PubMed (PMC or MEDLINE) journals have already shown high performance in their citation frequencies. The average cites per document (2 years) of 16 non-PubMed journals in the 2021 SCImago Journal Rank (SJR; https://www.scimagojr.com) was 0.731; while that of nine PubMed journals was 1.703 (Supplementary material 1), excluding seven journals for which the metrics were not counted yet in the 2021 SJR.

## Strategies to be promoted to a top-tier journal in the nursing category

Now that KJWHN will be searchable in PubMed and PMC, article quality offers its best chance to compete with other international journals. Therefore, the editor's job is to recruit high-quality manuscripts from researchers both in Korea and worldwide. What are high-quality manuscripts? A manuscript should be scientifically sound by adopting the following style and format: the study design should be specified; the hypothesis could be defined and answered; manuscripts should be described according to the appropriate reporting guidelines (https://www.equator-network.org/), for example, CONSORT for randomized controlled trials, STROBE for observational studies, PRISMA for systematic reviews and meta-analyses, and TREND for quasi-experimental studies, and COREQ for qualitative studies; statistical power should be verified through study size estimation [5]; and the interpretation of the results should not be overstated. KJWHN has already realized the above strategies to be scientifically sound, which is why PMC has accepted the journal. I trust that current and future editors will continue the high-quality peer review and editing.

KJWHN has adopted most publishing policies adequately by adhering to the *Principles of Transparency and Best Practice in Scholarly Publishing* (3rd version), like many other scientific journals in Korea [6]. The journal website also provides a top-tier user-friendly interface. I suggest a mandatory data-sharing policy to enhance other researchers’ use of shared data. Data sharing is currently optional, so not all data are shared, and data are usually available by contacting corresponding authors. Some authors have shared their research data in Harvard Dataverse (https://dataverse.harvard.edu/) [7,8]. It remains uncertain whether data sharing can provide an incentive for other researchers to use the shared data as their research materials, and there is still no evidence of the advantages of data sharing for further use by other researchers. However, mandatory data sharing should be pursued more actively to confirm the reproducibility of results.

**Figure 1.**
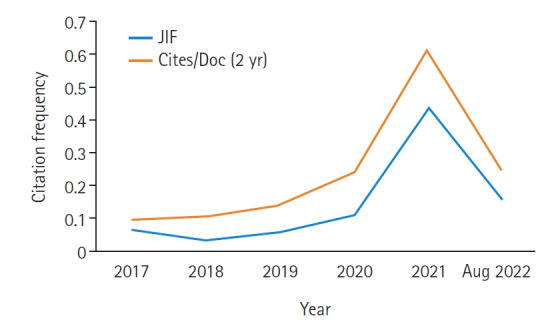
Changes in the Journal Impact Factor (JIF) and cites per document within 2 years (Cites/Doc [2 yr]) of the *Korean Journal of Women Health Nursing* manually calculated from Web of Science Core Collection and Scopus on August 9, 2022.

The Journal Impact Factor, manually calculated from Web of Science and cites per document (2 years) from Scopus, is presented in Figure 1. The values for KJWHN in 2021 were 0.44 and 0.60, respectively. This is a fantastic result, even though KJWHN was just recently indexed in PMC. Although these values are still not high enough for KJWHN to be a top-tier journal, the citation frequencies in both databases will soar soon, like other PMC journals in Korea [4].

## Fulfillment of the aims of the journal

KJWHN aims to be “a core resource for cutting-edge advancements and clinical applications of new nursing practice, therapeutic protocols for managing health problems in women, and innovative research on gender-based issues that impact treatment and nursing care.” By being indexed in PMC, the journal will be able to meet worldwide readers and nurses to provide them with invaluable nursing experiences. This is another way to complete the aims and mission of the journal. The past and present editors have done their best to refine manuscripts and publishing policies to reach the present accomplishments for the journal. I applaud them for their enormous and endless devotion and sacrifice for this academic society journal that is small, yet like a gem in its contribution to women’s health nursing in Asia.

## Supplementary materials

Further details on supplementary materials are presented online (available at https://doi.org/10.4069/kjwhn.2022.08.19).

Supplementary material 1. List of nursing journals indexed in international databases, including PubMed Central, MEDLINE, Scopus, SCIE, and ESCI).

## References

[1] Huh S (2021). PubMed Central as a platform for the survival of open-access biomedical society journals published in Korea. Sci Ed.

[2] Huh S (2022). Congratulations on Child Health Nursing Research becoming a PubMed Central journal and reflections on its significance. Child Health Nurs Res.

[3] Jeong GH, Huh S (2014). Increase in frequency of citation by SCIE journals of non-Medline journals after listing in an open access full-text database. Sci Ed.

[4] Kim S (2021). Korean Journal of Women Health Nursing is indexed in Scopus and stepping closer to international connectivity. Korean J Women Health Nurs.

[5] Kang H (2021). Sample size determination and power analysis using the G*Power software. J Educ Eval Health Prof.

[6] Choi YJ, Choi HW, Kim S (2020). Compliance of “Principles of transparency and best practice in scholarly publishing” in Korean academic society-published journals listed in Journal Citation Reports. Sci Ed.

[7] Kim E, Kim HW (2022). Nurses’ attitudes and stress related to perinatal bereavement care in Korea: a cross-sectional survey. Korean J Women Health Nurs.

[8] Jung YE, Sung MH (2022). Do parenting stress, work-family conflict, and resilience affect retention intention in Korean nurses returning to work after parental leave?: a cross-sectional study. Korean J Women Health Nurs.

