# Supplementary material for: Marking the inclusion of the Korean Journal of Women Health Nursing in PubMed Central and strategies to be promoted to a top-tier journal in the nursing category
Source: Korean J Women Health Nurs. 2022 Sep 30;28(3):165–8. doi: 10.4069/kjwhn.2022.08.19 (PMC9619161; doi:10.4069/kjwhn.2022.08.19)
Supplement: Supplementary material 1. — List of nursing journals indexed in international databases, including PubMed Central, MEDLINE, Scopus, SCIE, and ESCI). [file kjwhn-2022-08-19-suppl.pdf]

KJWHN

Supplementary material 1. List of nursing journals indexed in international databases, including PubMed Central, MEDLINE, Scopus, SCIE, and ESCI).

Journal title	Owner	Language	country	NLM status	Scopus	Cites/Doc. (2years) 2021	SCIE/ESCI	JIF 2021
International journal of community based nursing and midwifery	Fatemeh School of Nursing & Midwifery	English	Iran	PMC, MEDLINE	Scopus	2.529		
Korean Journal of Women Health Nursing	Korean Society of Women Health Nursing	Korean, English	Korea	PMC	Scopus	0.6		
Florence Nightingale journal of nursing	İstanbul Üniversitesi Florence Nightingale Faculty of Nursing	English	Turkey	PMC	Scopus		ESCI	
Child health nursing research	Adong Kanho Hakhoe	English	Korea	PMC	Scopus	0.72		
International journal of nursing sciences	Chinese Nursing Association	English	China	PMC	Scopus	3.3	ESCI	
Journal of caring sciences	Nursing & Midwifery Faculty, Tabrīz University of Medical Science	English	Iran	PMC	Scopus			
Iranian journal of nursing and midwifery research	Isfahan University of Medical Sciences	English	Iran	PMC	Scopus	1.38	ESCI	
Asian Nursing Research	Korean Academy of Nursing	English	Korea	MEDLINE	Scopus	2.929	SCIE	2.612
Journal of Nursing Research	Taiwan hu li xue hui	English	Taiwan	MEDLINE	Scopus	2.15	SCIE	2.517
Japan Journal of Nursing Science	Japan Academy of Nursing Science	English	Japan	MEDLINE	Scopus	1.845	SCIE	1.691
Journal of Korean Academy of Nursing	Korean Academy of Nursing	Korean, English	Korea	MEDLINE	Scopus	1.19	SCIE	1.277
Journal of Nursing	Taiwan Nurses Association	Chinese	Taiwan	MEDLINE	Scopus	0.39		
Journal of Nursing and Midwifery Sciences	Mazandaran University of Medical Sciences	English	Iran		Scopus		ESCI	
Asia-Pacific Journal of Oncology Nursing	Asia Oncology Nursing Society	English	Hong Kong		Scopus	1.99	SCIE	2.220
Nursing Practice Today	Tehran University of Medical Sciences	English	Iran		Scopus	1.092		
Journal of Korean Academy of Nursing Administration	Korean Academy of Nursing Administration.	Korean, English	Korea		Scopus	0.89		
Nursing and Midwifery Studies	Kashan University Medical Sciences	English	India		Scopus	0.83	ESCI	
Belitung Nursing Journal	Belitung Raya Foundation	English	Indonesia		Scopus	0.82	ESCI	
Pacific Rim International Journal of Nursing Research	Thailand Nursing & Midwifery Council	English	Thailand		Scopus	0.75	ESCI	
Journal of Korean Academic Society of Nursing Education	Korean Academic Society of Nursing Education	Korean, English	Korea		Scopus	0.74		
Korean Journal of Adult Nursing	Korean Society of Adult Nursing	Korean, English	Korea		Scopus	0.74		
Journal of Korean Gerontological Nursing	Korean Gerontological Nursing Society.	Korean, English	Korea		Scopus	0.71		
Journal of the Korean Academy of Fundamentals of Nursing	Korean Academy of Fundamentals of Nursing	Korean, English	Korea		Scopus	0.58		
Nurse Media Journal of Nursing	Diponegoro University- Department of Nursing, Faculty of Medicine	English	Indonesia		Scopus	0.56		
Journal of Holistic Nursing and Midwifery	Guilan University of Medical Sciences	Persian	Iran		Scopus	0.54		
Journal of Korean Academy of Community Health Nursing	Korean Academy of Community Health Nursing	Korean, English	Korea		Scopus	0.51		
Journal of Psychiatric Nursing	Psychiatric Nurses Association,	English	Turkey		Scopus	0.494		
HAYAT: Ulūm-i Pizishkī va Khadamāt-i Bihdāshtī, Darmānī-i Tihrān.	Tehran University of Medical Sciences Tehran University of Medical Sciences	Persian	Iran		Scopus	0.347		
Philippine Journal of Nursing	Philippines Nurses Association	English	Philippines		Scopus	0.11		

(Continued to the next page)

Supplementary material 1. Continued

Journal title	Owner	Language	country	NLM status	Scopus	Cites/Doc. (2years) 2021	SCIE/ESCI	JIF 2021
Chinese Journal of Practical Nursing	Editorial Board of "Chinese Journal of Practical Nursing"	Chinese	China		Scopus			
Journal of Korean Academy of Psychiatric and Mental Health Nursing	Korean Academy of Psychiatric and Mental Health Nursing	Korean, English	Korea		Scopus			
Jurnal Keperawatan Indonesia	Universitas Indonesia	Indonesian, English	Indonesia		Scopus			
Asian Oncology Nursing	Korean Oncology Nursing Society	Korean, English	Korea		NO		ESCI	
				16	non-pubmed	0.7314375		
				11	PubMed	1.7033		

https://doi.org/10.4069/kjwhn.2022.08.19
